# Altered Cerebellar-Cerebral Functional Connectivity in Geriatric Depression

**DOI:** 10.1371/journal.pone.0020035

**Published:** 2011-05-26

**Authors:** Emmanuel Alalade, Kevin Denny, Guy Potter, David Steffens, Lihong Wang

**Affiliations:** 1 Morehouse School of Medicine, Atlanta, Georgia, United States of America; 2 Brain Imaging and Analysis Center, Duke University Medical Center, Durham, North Carolina, United States of America; 3 Department of Psychiatry and Behavioral Sciences, Duke University Medical Center, Durham, North Carolina, United States of America; The University of Melbourne, Australia

## Abstract

Although volumetric and activation changes in the cerebellum have frequently been reported in studies on major depression, its role in the neural mechanism of depression remains unclear. To understand how the cerebellum may relate to affective and cognitive dysfunction in depression, we investigated the resting-state functional connectivity between cerebellar regions and the cerebral cortex in samples of patients with geriatric depression (n = 11) and healthy controls (n = 18). Seed-based connectivity analyses were conducted using seeds from cerebellum regions previously identified as being involved in the executive, default-mode, affective-limbic, and motor networks. The results revealed that, compared with controls, individuals with depression show reduced functional connectivity between several cerebellum seed regions, specifically those in the executive and affective-limbic networks with the ventromedial prefrontal cortex (vmPFC) and increased functional connectivity between the motor-related cerebellum seed regions with the putamen and motor cortex. We further investigated whether the altered functional connectivity in depressed patients was associated with cognitive function and severity of depression. A positive correlation was found between the Crus II–vmPFC connectivity and performance on the Hopkins Verbal Learning Test-Revised delayed memory recall. Additionally, the vermis–posterior cinglate cortex (PCC) connectivity was positively correlated with depression severity. Our results suggest that cerebellum–vmPFC coupling may be related to cognitive function whereas cerebellum–PCC coupling may be related to emotion processing in geriatric depression.

## Introduction

Depression has been modeled as a failure of the coordination between the dorsal cognitive control system and the ventral emotional system [1]. While numerous neuroimaging studies support the essential role of the prefrontal-striato-limbic circuits in depression [1], [2], [3], there are also a number of reports indicating an altered neural response in the cerebellum [4], such as increased cerebellar-vermal blood flow in depressed patients with cognitive impairment [5], reduced cerebellar volume during depressed state [6], [7], and progressively reduced cerebellar volume over time [8]. Although there is clear evidence of the involvement of the cerebellum in depression [4], [9], the functional role of the cerebellum in depression remains unclear.

There is ample evidence that the cerebellum not only subserves motor function, planning, and coordination of movement, but also plays an important role in emotion and cognition [10]. Recent reviews have paid much attention to the involvement of the cerebellum in emotion and cognition [11], [12]. Anatomically, regions of the cerebellum, such as the vermis, fastigial nucleus, and flocculonodular lobe, have reciprocal connections with brainstem reticular nuclei [13], [14] and regions in the limbic and autonomic system, including hypothalamus [15], [16], [17], ventral tegmental area, periaqueductal gray [18], hippocampus, and amygdala [19]. The cerebellum also receives projections from the rostral and caudal anterior cingulate through the pons [20]. These connections may provide an anatomical basis for the cerebellum's role in emotion. Connections between the prefrontal cortex and the cerebellum have also been found, which include descending projections from the dorsolateral and dorsomedial prefrontal cortex to the cerebellum through the medial pons and ascending projects from cerebellum through thalamus to prefrontal cortex. These connections are hypothesized to be the neural substrates for the cognitive function of the cerebellum [9], [10], [21]. In animal studies, electrical stimulation of the vermis area of the cerebellum evoked responses in the orbitofrontal cortex, anterior cingulate, amygdala, and hippocampus [19], [20], [22]. In human studies, stimulation of the surface of cerebellum through the implantation of electrodes revealed alleviation of depression [19]. The cognitive affective syndrome in patients with cerebeller lesions reported by Schmahmann and Sherman [23] provides strong evidence of the involvement of the cerebellum in emotion and cognition. In addition, response to emotional stimuli in the cerebellum, particularly in the vermis, has been found in a number of neuroimaging studies [24], [25]. These studies provide empirical evidence supporting an essential contribution of the cerebellum to the affective and cognitive dysfunction in depression.

Recent use of intrinsic resting-state functional connectivity enables us to understand the functional connectivity between the cerebellum and the cerebrum [26]. Using an independent component analysis (ICA) approach, Habas and colleagues [27] identified regions in the cerebellum that ‘belong’ to the dorsal executive, salience, default-mode, and sensorimotor networks, separately. Meanwhile, using seeds from these four neural networks in the cerebral cortex, Krienen and Buckner [26] also investigated the regions in the cerebellum that are functionally connected with the dorsal executive, default-mode, affective, and motor networks of the cerebrum. They found that the lateral hemisphere of the cerebellum was functionally connected with the dorsolateral prefrontal cortex (dlPFC), suggesting its potential involvement in executive function. Additionally, the Crus I of the cerebellum was functionally connected with the medial prefrontal cortex and anterior cingulate indicating its involvement in default-mode activity and emotional processing [26]. These studies of functional connectivity between the cerebellum and cerebrum provide a topographic functional map of the cerebellum, which could explain the role of cerebellar volumetric [6], [28], [29], [30] and activation changes [5], [31], [32] in depression. However, to our knowledge, there is no direct evidence showing how the relationship of the frontal-cerebellar connectivity with mood or cognitive function is altered in depression.

Using seeds in the cerebellum that were suggested to be involved in emotional and cognitive function by Krienen and Buckner [26], we compared the intrinsic functional connectivity between the cerebellum and the cerebrum in the executive, default-mode, affective-limbic, and motor networks in depressed patients and healthy controls. Given that cognitive impairment is frequently seen in geriatric depression, this study focused on older individuals to investigate the association of altered connectivity with severity of depression, memory, and executive functions. We also examined the cerebellum-cerebrum functional connectivity in older healthy individuals to validate previous network findings in older adults.

We hypothesized that depressed patients would have decreased functional connectivity between the executive network-related regions in the cerebellum and the prefrontal cortex, and they would have increased connectivity between the affective-limbic network-related regions in the cerebellum and affective regions including the amygdala or ventral striatum. We also hypothesized that the alterations in the connectivity from the affective-limbic network-related regions of the cerebellum to the cerebrum would be associated with severity of depression.

## Materials and Methods

### Participants

Twenty-nine individuals participated in this study (11 depressed, 18 non-depressed controls). Participants were recruited from the Conte Center for the Neuroscience of Depression in Late Life at Duke University Medical Center. All depressed patients met DSM-IV criteria for major depression. They were either in an active major unipolar depressive episode or at least had some depressed symptoms, with a Montgomery-Åsberg Depression Rating Scale (MADRS) [33] score of 8 or more at the time of participation in the study. The exclusion criteria for depressed subjects included: (1) another major psychiatric illness, including bipolar disorder, schizophrenia, or dementia; (2) alcohol or drug abuse or dependence; (3) neurological illness, including dementia, stroke, and epilepsy; (4) medical illness, medication use, or disability that would prevent the participant from completing neuropsychological testing; and (5) contraindications to MRI. All non-depressed subjects were cognitively intact, had no history or clinical evidence of dementia, and all scored 28 or more on the Mini-Mental State Examination. Among the 11 depressed participants, 6 were receiving antidepressant monotherapy (3 on an SSRI, 2 on venlafaxine, and 1 on amitriptyline) and 5 were receiving combination treatment (4 on SSRI combined with either SNRI, SARI, or DNRI and 1 on SSRI, NDRI, and SNRI).

Prior to the fMRI, subjects' cognitive function was assessed using a short, 30-minute battery of neuropsychological tests. The neuropsychological tests included the Mini-Mental State Exam (MMSE), Category Fluency (Vegetable Naming), Hopkins Verbal Learning Test-Revised (HVLT-R), Immediate and Delayed Story Recall from the Rivermead Behavioral Memory Test, Trail Making Test (Trail A and Trail B), WAIS-III Digit Span, WAIS-III Digit–Symbol Substitution Test (DSST), and Stroop Color and Word Test. The study received ethics committee approval by Duke School of Medicine Institutional Review Board and, after being explained the purpose and procedures to be used in the study, all subjects gave verbal and written consent.

### Neuroimaging Acquisition

We obtained a 5-minute resting fMRI scan for each participant. Participants were instructed to rest without moving, keep their eyes open, and focus on a fixation cross presented in the center of the screen inside the scanner. All participants were scanned using a research-dedicated 3.0 T GE EXCITE HD scanner (GE Medical Systems, Milwaukee, Wisconsin). Oblique spoiled gradient-recalled acquisition images (three-dimensional, whole-brain) were acquired parallel to the anterior commissure (AC) - posterior commissure (PC) plane for high-resolution T1-weighted structural images with a matrix of 256×256×169, slice thickness of 1 mm. Inward spiral sequence functional images were acquired with the following parameters: TR = 2000 ms, TE = 31 ms, FOV = 24 cm, flip angle  = 90°, matrix  = 64×64×34, slice thickness  = 3.75 mm with 3.75 mm^3^ isotropic voxels.

### Data Analyses

FEAT (FMRI Expert Analysis Tool) Version 5.98, part of the FSL analysis package (fMRIB's Software Library, www.fmrib.ox.ac.uk/fsl), was used to conduct the standard image pre-processing procedures including slice-timing alignment, motion correction, coregistration, non-brain voxel extraction, normalization, and smoothness (6 mm^3^ kernel). Temporal filtering settings were applied using a high-pass filter (Gaussian-weighted least-squares straight line fitting, with sigma  = 100.0 s) and a low-pass filter (Gaussian low-pass temporal filtering: HWHM 2.8 s) following Biswal and colleagues [34].

To identify the functionally-connected networks between the cerebellum and cerebrum, seed-based correlation analyses were carried out by extracting the time series from regions of interest (ROI) using FSL's FLIRT. Seeds that were shown to have a fronto-cerebellar connection from Krienen and Buckner [26] and Stoodley and Schmahmann [35] were used to identify executive, default-mode, affective-limbic, and motor networks in the cerebellum. For the executive network, three pairs of bilateral seeds were chosen: Crus I_Exec1_, Crus II_Exec2_ and Lobule VI_antExec_. Both Crus I_Exec1_ and Crus II_Exec2_ have been shown to be functionally connected with the posterior region of the dlPFC by Krienen and Buckner [26]. Lobule VI_antExec_ was shown to be functionally coupled with the anterior portion of the dlPFC [26]. For the default-mode network, bilateral Crus I_DMN_ seeds were selected, which were found to have functional connections to default-mode network (DMN) regions [26]. We used bilateral Lobule VI_Limbic_ and the left Vermis_Limbic_ for the affective-limbic network. These regions of the cerebellum were found to be activated during emotional processing [35]. For the motor-network regions, bilateral Lobule V_Motor_ seeds were used and previously found to be functionally connected to the motor cortex [35]. In Table 1, these seed regions are grouped by network with their center coordinates listed. A 5-mm radius sphere was drawn from each center point as an ROI. The timecourse during the 5-minute resting scan within the sphere was extracted. The timecourse of each ROI was then entered as a regressor into the first-level (within-subject) general linear model (GLM) using FEAT. Nuisance regressors (global signal, white matter, and motion parameters) were also entered into the model.

**Table 1:** Cerebellar Regions of Interest (seeds) and Coordinates Grouped By Network.

Cerebellar Network	MNI (x,y,z)
***Executive Network***	
L Crus I_**Exec1**_	−12, −78, −28
R Crus I_**Exec1**_	12,−78,−28
L Crus II_**Exec2**_	−36,−70,−46
R Crus II_**Exec2**_	36, −68, −44
L Lobule VI_**antExec**_	−36, −52, −34
R Lobule VI_**antExec**_	36, −52, −34
***Default-Mode Network***	
L Crus I_**DMN**_	−32, −76, −34
R Crus I_**DMN**_	34, −80, −36
***Affective-Limbic Network***	
R Lobule VI_**Limbic**_	26, −64, −34
L Lobule VI_**Limbic**_	−26, −64, −34
L Vermis_**Limbic**_	−4, −80, −34
***Motor Network***	
R Lobule V_**Motor**_	22 −52 −22
L Lobule V_**Motor**_	−20 −50 −24

To examine group differences for each region's seed-based functional connectivity, we used a mixed-effects (FLAME 1) two-sample t-test analysis on each seed-based connectivity map. To test the relationship between the functional connectivity and individual variation in severity of depression, we conducted a regression analysis in the depressed group using participants' MADRS scores as a regressor. To test the relationship between the functional connectivity and individual variation in memory performance and executive function, we conducted a regression analyses using HVLT-R delay scores and Stroop Color and Word test scores from all participants, including both patients and controls, as covariates in separate third-level models. Given the age difference between the two groups, we used age as a regressor to remove any age effect from the third level analyses. Statistical results used a voxel significance threshold of z>2.3 and a whole-brain-corrected cluster-significance threshold of p<0.05 [36]. Significant clusters were selected as ROIs for the regression plots to double confirm the voxel-based whole-brain analysis.

## Results

### Clinical Profile of the Participants

Demographic details of the participants and performance on the memory and executive function-related tests from the neuropsychological battery are listed in Table 2. In summary, there were 11 females and 7 males in the control group whereas there were 10 females and 1 male in the depressed group (chi square = 3.04, df = 1, p = 0.08). The depressed group was significantly younger in age than the control group (two-sample t-test, t_26_ = 3.05, p = 0.005). Therefore, age was used as a covariate in data analyses to remove any age effect.

**Table 2:** Participant Demographic and Neuropsychological Testing Data.

	Control N = 18	Depressed N = 11	*P* values
Age (SD)	71.2 (6.6)	64.9 (4.5)	0.005
No. of Female/Male	11/7	10/1	
Education (SD) years	15.8 (2.3)	14.4 (3.1)	0.192
MADRS (SD)	0.4 (0.8)	17.5 (8.2)	<0.001
No. with early/late onset depression	N/A	7/4	
Age of onset (SD)	N/A	33.5 (18.5)	
Duration of illness in years (SD)	N/A	31.3 (17.9)	
No. with multiple depressive episodes	N/A	8	
No. taking antidepressants	N/A	11	
MMSE (SD)	29.2 (1.2)	28.9 (1.7)	0.605
Memory function			
*Rivermead Delay(SD)*	7.5 (3.0)	3.9 (3.1)	0.011
*HVLT Delay(SD)*	10.1 (1.3)	6.0 (4.3)	0.021
Executive function			
*Digit span (SD)*	14.1 (4.5)	11.6 (3.0)	0.095
*Symbol-Digit Modality (SD)*	70.1 (11.3)	53.8 (16.7)	0.021
*Stroop Color-Word task (SD)*	34.1 (4.6)	30.7 (8.2)	0.267

The mean (SD) depression severity score measured by the MADRS for the depressed group was 17.5 (8.2), which was significantly greater than the control group (two-sample t-test, t_26_ = 6.85, p<0.001). Among the 11 depressed patients, all were on multiple or mono- antidepressant therapy. Although the MMSE was not significantly different between the depressed and the control group (two-sample t-test, t_26_ = 0.44, p = 0.605), the depressed group revealed significantly poorer performance on the neuropsychological tests, including the two memory tests—the HVLT-R delay (two-sample t-test, t_26_ = 2.49, p = 0.021) and Delayed Story Recall from the Rivermead Behavioral Memory Test (two-sample t-test, t_26_ = 2.86, p<0.011), and one of the executive tests—the DSST(two-sample t-test, t_26_ = 2.64, p = 0.021).

### The Cerebellar-Cerebral Functional Connectivity in Healthy Older Subjects

**Figure 1:**
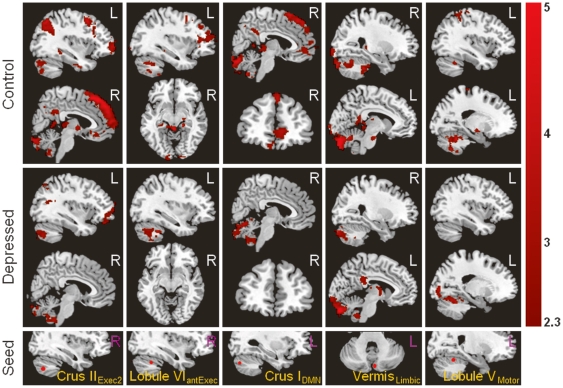
Resting-state functional connectivity of cerebellum seed regions (row five) with cerebral regions from the healthy control group (rows one and two) and depression group (rows three and four). The significant functional connectivity maps shown in each column were computed from the cerebellar seed region shown in the bottom row. The seed regions shown here include: Crus II_Exec2_, the Crus II region found to be functionally connected with a cerebral executive region (the posterior dlPFC) in previous studies; Lobule VI_antExec,_ the Lobule VI region found to be functionally connected with a cerebral executive region (the anterior dlPFC) in previous studies; Crus I_DMN_, the Crus I region found to be functionally connected with the default-mode network (mPFC) in previous studies; Vermis_Limbic_, the vermis region found to be functionally connected with a limbic region (ACC); Lobule V_Motor_, the Lobule V region found to be functionally connected with the motor cortex.

Our study on cerebellar-cerebral functional connectivity in healthy elderly subjects largely replicated the findings of Krienen and Buckner in younger subjects [26]. The executive network region within the cerebellum (right Crus II_Exec2_ and right Lobule VI_antExec_) did show connectivity with the executive network in the cerebral cortex, specifically the contra-lateral dlPFC and the inferior parietal cortex (Figure 1). Interestingly, the significant functional coupling between the cerebellum and dlPFC was only found in the right cerebellar seed and left dlPFC, but not with the cerebellar seeds in the left hemisphere. In addition, the executive network regions within the cerebellum (specifically right Crus II_Exec2_) also showed functional connectivity with the putamen and default-mode network regions including the posterior cingulate cortex (PCC) and the ventral medial prefrontal cortex (vmPFC).

The seed within the default-mode network (DMN) in the cerebellum, left Crus I_DMN_, showed functional connectivity with default-mode network regions in the cerebrum including the ventromedial prefrontal cortex (vmPFC), dorsomedial prefrontal cortex (dmPFC), and the PCC (Figure 1). The right Crus I_DMN_, however, did not show significant functional connectivity with the default-mode network regions. Instead, it showed functional connectivity with the caudate, thalamus, and fusiform gyrus when using a relatively lenient threshold on cluster correction—z>2 rather than z>2.3—and a cluster corrected significance threshold of p = 0.05.

The affective-limbic network of the cerebellum, specifically bilateral Lobule VI_Limbic_ and left Vermis_Limbic_, demonstrated functional connectivity with the PCC, inferior parietal cortex, brainstem, thalamus, and hypothalamus in addition to regions within the cerebellum (Figure 1).

The cerebellar motor network seed regions demonstrated functional connectivity with the sensorimotor regions including the cerebellum, medial lemnis fasiculus in the pons, bilateral red nucleus in the brainstem, right putamen, right thalamus, bilateral sensory cortex, and anterior cingulate cortex (ACC) (Figure 1).

### Decreased Cerebellar-Cerebral Functional Connectivity in Depressed Patients Relative to Healthy Control Subjects

**Figure 2:**
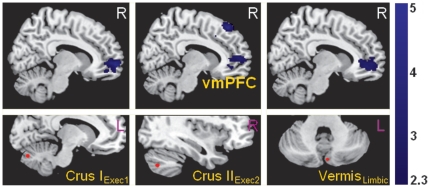
Significantly reduced functional connectivity in depressed patients between cerebellar executive and affective-limbic seed regions (shown in the lower row) with the ventromedial prefrontal cortex (vmPFC).

Compared with healthy control subjects, the depressed group showed significantly reduced functional connectivity between the vmPFC and several cerebellar seed regions from the executive and affective-limbic networks, specifically bilateral Crus I_Exec1_ and right Crus II_Exec2_ seeds, as well as left Vermis_Limbic_ (Figure 2).

**Figure 3:**
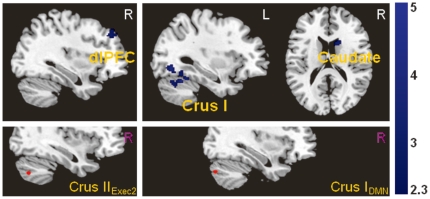
Significantly reduced functional connectivity in depressed patients between cerebellar executive and default-mode seed regions (shown in the lower row) with cerebral areas.

In addition, the depressed group showed reduced functional coupling relative to controls between the executive region in the cerebellum and the executive regions in the cerebral cortex, specifically between the right Crus II_Exec2_ seed and the right dlPFC and dmPFC (BA8) (Figure 3). The executive seed regions left Crus II_Exec2_ and Lobule VI_antExec_ also showed reduced connectivity with other regions within the cerebellum.

The default-mode network seed in the cerebellum, specifically right Crus I_DMN_, showed decreased connectivity with the right head of the caudate, right insula/putamen, left fusiform gyrus, and left Lobule VI in depressed patients relative to controls (Figure 3).

For the affective-limbic network in the cerebellum, depressed patients had reduced functional connectivity relative to controls between the left Lobule VI_Limbic_ with the right inferior parietal cortex (BA39) and the PCC and the left Vermis_Limbic_ with the left ventrolateral prefrontal cortex (vlPFC, BA47).

Interestingly, the left Lobule V_Motor_ seed showed decreased connectivity with the dorsal executive regions in the cerebral cortex—the left dlPFC and vlPFC—in patients.

All regions showing decreased connectivity with cerebellar seeds in depressed patients relative to controls are listed in Table 3.

**Table 3:** Brain Regions Showing Decreased Cerebeller-Cerebral Connectivity in Geriatric Depression Compared With the Control Group.

Cerebellar Seed Region	Regions showing decreased connectivity in geriatric depression	BA	Side	Voxel size	MNI Coordinates (x,y,z)	Z value
***Executive Network***						
R Crus I_Exec1_	mPFC	BA10	R	765	10, 50, 0	3.75
	vmPFC/rACC	BA32	R		14, 42, 2	3.63
L Crus I_Exec1_	vmPFC	BA10	R	500	12, 60, −2	4.51
R CrusII_Exec2_	vmPFC/rACC	BA32	R	485	14, 44, 6	4.27
	dmPFC	BA8	R	691	6, 38, 52	4.59
	dlPFC	BA9	R	490	28, 40, 38	4.44
L Crus II_Exec2_	Lobule VI		R	910	30, −56, −24	3.64
	Vermis		L		−2, −46, 10	3.96
	Brainstem (red nuclus)				0, −24, −8	3.61
L LobuleVI_antExec_	Lobule VI		R	67	0,−72,−14	3.58
	Crus I		R	82	24,−78,−26	3.25
	Crus I		L	86	−8,−74,−24	3.48
***Default-Mode Network***						
R Crus I_DMN_	Caudate		R	83	16, 12, 16	3.55
	Putamen		R	72	30, 10, −6	4.13
	Fusiform Gyrus	BA19	L	84	−22, −54, −14	4.27
	Lobule VI		L	539	−32, −56, −24	4.38
***Affective-Limbic Network***						
L Vermis_Limbic_	vmPFC/mPFC	BA10	R	517	16, 46, 6	3.62
	vlPFC	BA47	L	474	−44, 26, 4	4.09
L Lobule VI_Limbic_	mPFC	BA10	R	660	8, 62, 30	3.99
	Precuneus/PCC	BA23	R	1096	8, −50, 24	3.76
	Inferior parietal cortex	BA39	R	632	50, −58, 22	4.32
***Motor Network***						
L Lobule V_Motor_	vlPFC	BA47	L	1301	−50 ,28, 2	3.84
	dlPFC	BA10	L		−40, 58, 2	3.48

### Increased Cerebellar-Cerebral Functional Connectivity in Depressed Patients Relative to Healthy Control Subjects

**Figure 4:**
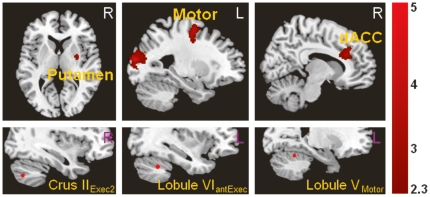
Significantly increased functional connectivity in depressed patients between cerebellar executive and motor seed regions (shown in the lower row) with cerebral areas.

Within the executive network in the cerebellum, a number of seeds showed increased connectivity with other networks in depressed patients, predominantly with the self-referential processing-related region, the dmPFC, or interoceptive cortex, the insula [37]. Specifically, the right Crus II_Exec1_ displayed increased connectivity with the dmPFC (BA10) in patients relative to controls. A similar pattern of increased connectivity in patients was seen in the right Crus II_Exec1_ with the insula and the right Crus II_Exec2_ also with the insula. The Crus II_Exec2_ also showed increased connectivity with the motor network, specifically the putamen; and the left Lobule VI_antExec_ showed increased connectivity with the supplementary motor area as well as the middle temporal cortex (Figure 4).

The left Lobule V_Motor_ showed increased functional connectivity with the right dorsal ACC/dmPFC (Figure 4). All regions showing increased connectivity with cerebellar seeds in depressed patients relative to controls are listed in Table 4.

**Table 4:** Brain Regions Showing Increased Cerebeller-Cerebral Connectivity in Geriatric Depression Compared With the Control Group.

Cerebellar Seed Region	Regions showing increased connectivity in geriatric depression	BA	Side	Voxel size	MNI Coordinates (x,y,z)	Z value
***Executive Network***						
R Crus I_Exec1_	dmPFC	BA10	L	481	−18,60,18	4.51
	Insula	BA13	L	3497	−44, −12,18	5.14
R Crus II_Exec2_	Insula	BA13	R	695	44, −34, 22	3.66
	Putamen		R		30, −2, 6	3.14
L Lobule VIant_Exec_	Precentral cortex	BA6	L	446	−42, 0, 46	4.42
	Middle Occipital	BA19	L	685	−26, −90, 16	4.5
***Affective-Limbic Network***						
L Lobule VI_Limbic_	Middle temporal	BA39	L	1029	−34, −50, 6	3.67
	Motor Cortex	BA2	L	2056	−42, −36, 62	6.96
***Motor Network***						
L Lobule V_Motor_	dmPFC/dACC		R	762	20, 28, 36	3.75

### Correlation with Cognition and Affect

**Figure 5:**
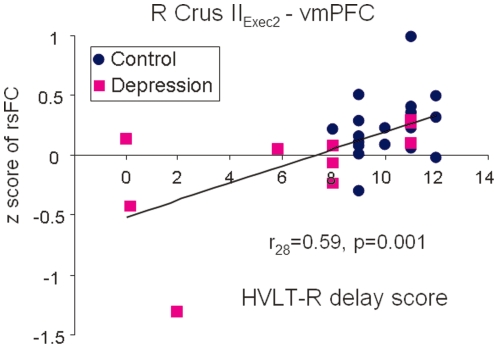
Significant correlation of decreased right Crus II_ Exec2_-vmPFC connectivity with poorer performance on the HVLT-R delay across both depressed (purple squares) and control subjects (blue dots). Data from all participants were included in this regression analysis.

**Figure 6:**
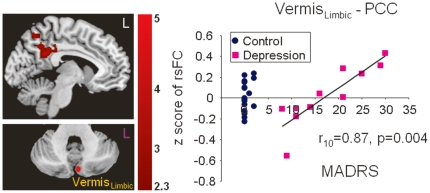
Significant correlation of increased Vermis_Limbic_- PCC connectivity with severity of depression symptoms in the depressed group (purple squares). Data from the depressed patients only were included in this regression analysis.

We further investigated whether any of the significant differences in the resting state functional connectivity between depressed patients and controls were correlated with scores on memory and executive function tasks or with severity of depression. Using scores for each participant on the HVLT-R delay test and the Stroop Color and Word test as regressors in separate third-level group analysis models, we found a positive correlation between the right Crus II_Exec2_–vmPFC connectivity and performance on the HVLT-R delay (r_27_ = 0.59, p = 0.001) across all subjects (Figure 5). Given that the majority of control subjects had a MADRS score of 0, the correlation between the functional connectivity with severity of depression was conducted only in the depressed group. Within the depressed group, the Vermis_Limbic_–PCC coupling was positively correlated with severity of depression measured by the MADRS (r_10_ = 0.87, p = 0.004) (Figure 6).

## Discussion

We found aberrant functional connectivity in geriatric depression patients between the cerebellum and the cerebral cortex in several neural networks. The cerebellar seed regions in both the executive and affective-limbic networks revealed decreased functional connectivity with the vmPFC. Furthermore, the decreased Crus II–vmPFC connectivity was correlated with poorer memory performance (HVLT-R delay) confirming a role of the Crus II–vmPFC connectivity in cognition. In addition, there was a significant correlation between the Vermis–PCC connectivity and severity of depression, which supports the involvement of the cerebellum in emotional processing. Therefore, our results provide strong support for the contribution of the cerebellum to both cognitive and affective dysfunction in depression.

The vmPFC has wide connections with both affective-limbic regions—such as the amygdala, hippocampus, and hypothalamus—and with executive control and emotional regulation regions—such as the lateral orbital frontal cortex (OFC), dlPFC, vlPFC, and dorsal ACC. It possibly connects to the cerebellum through the cingulate-pontine-cerebellar pathway [38]. Several studies have reported the important role of the vmPFC in emotional regulation [39], extinction memory [40], and self-referential processing [35]. Together with the dmPFC, PCC, and hippocampus, the vmPFC has also been proposed as a node of the affective appraisal network [41] and the vmPFC showed a preference for positive emotional experiences [42]. Consistent with its key role in both cognition and emotion, we found decreased functional coupling between both cerebellar regions related to executive and affective-limbic networks with the vmPFC in depression. Furthermore, across both controls and depressed patients, we found the Crus II_Exec2_–vmPFC connectivity was positively correlated with the performance on the HVLT-R delay test, which highlights the importance of the functional coupling between the two regions in memory.

In addition to the decreased connectivity with the vmPFC, cerebeller seeds showed decreased connectivity within the executive network—the Crus II_Exec2_–dlPFC coupling—and decreased connectivity within the affective-limbic network—the Vermis_Limbic_–vlPFC coupling. The vlPFC has consistently been found to be involved in emotional regulation and activated during emotional reappraisal [43], [44], supporting the notion that the Vermis_Limbic_ indeed could be related to affective regulation. Consistently, a similar result was found in the study of Frodl and colleagues who reported reduced orbitofrontal cortex (OFC) and cerebellum coupling during negative emotional processing in younger patients with depression [45]. Interestingly, higher OFC-cerebellum connectivity was found in antidepressant non-responders [46]. Meanwhile, the Vermis_Limbic_–PCC coupling was positively correlated with severity of depression. The PCC is one of the key nodes of the default-mode network and the affective appraisal network. Consistent with previous findings of hyper default activity in the subgenual cingulate and thalamus area, here we also showed increased Vermis_Limbic_–PCC coupling, which could possibly represent heightened rumination during resting state, and decreased Vermis_Limbic_–vlPFC coupling as possible weakened emotion regulation in depressed patients.

The default-mode region of the cerebellum showed reduced connectivity with the caudate and ventral putamen. This result is in alignment with a recent study that showed reduced functional connectivity between the default-mode network and the caudate [47]. The caudate is part of the reward system and typically activated by motivation or reward tasks [48]. Lack of motivation and reduced response to reward is one of the core deficits in depression [49]. Reduced functional coupling between the default-mode network and reward system might reflect anhedonia, a state in depressed patients marked by a habitual lack of happiness or motivation.

The motor network region of the cerebellum, specifically the left Lobule V_Motor_, had reduced connectivity with the attention-executive network, the left dlPFC, which might be related to the psychomotor retardation in geriatric depression. However, the left Lobule V_Motor_ also showed increased connectivity with another attention-executive region, the right dorsal ACC. Interestingly, we also found increased connectivity between other cerebellar seeds and the motor network including the left Lobule VI_antExec_ with the supplementary motor cortex and Lobule VI_Limbic_ with the motor cortex. The increased connectivity between these regions could represent compensatory pathways related to the reduced coupling of Lobule V_Motor_–dlPFC, right Crus II_Exec2_–vmPFC, and Lobule VI_Limbic_–vmPFC.

Overall, by replicating the findings of Krienen and Buckner [26] in younger, healthy individuals, our study in a healthy older population confirmed the involvement of the cerebellum in executive, affective-limbic, default-mode, and motor networks of the cerebrum. Limitations of our study include the small sample size of the geriatric depression group and potential antidepressant effects. Future studies in a larger sample of geriatric depression patients free of medication to further investigate the causal relationship among the networks would further enhance our understanding of the role of the cerebellum in depression.

In summary, we found abnormal cerebellum-cerebral couplings in cognitive, default-mode, affective-limbic, and motor networks in geriatric depression patients. Therefore, by extending previous findings, our study has provided possible neural mechanisms for the involvement of the cerebellum in depression. Adding the cerebellum into the model of depression can stimulate the development of alternative interventions in depression, such as exercise or transcranial magnetic stimulation (TMS) of the cerebellum to modulate the altered cerebellar-cerebral pathways. Exercise has been shown to improve mood [50], expedite the recovery of depressed patients [51], and improve motor coordination skills and cognition [52]. While exercise can have many effects such as improving vascular endothelial function and blood flow [53], elevating the concentration of neurotransmitters such as serotonin and norepinephrine [54], increasing generation of BDNF (Brain-Derived Neurotrophic Factor), and promoting neurogenesis [55], [56], [57] and increasing the number of dendrite connections between neurons, it is possible that frequent exercise can also improve the efficiency of neural functional connectivity across all networks including the cerebellar-cerebral connectivity. Therefore, the altered functional cerebellar-cerebral connectivity found in our study can, potentially, be a target for monitoring the effect of exercise in geriatric depression. Future studies in depression to investigate the association between improved cognition and reduced depression symptoms with changes in the functional connectivity of the cerebellum would further enhance our understanding of the affective and cognitive function of the cerebellum.
